# Chicago Veterinary Society

**Published:** 1897-02

**Authors:** Lawrence Campbell

**Affiliations:** Secretary


Secretary.

### CHICAGO VETERINARY SOCIETY.

IN accordance with the votes of the members present at the last meeting of this Society, a banquet was held on January 14th, at the Sherman House, Chicago, at which the following members of the Society were present: Drs. Walker, George E. McEvers, J. G. Fish, Ryan, Gysel, Campbell, Bond, P. Quitman, E. L. Quitman, Roberts, Schwarzkopf, Hughes, Merillat, Flowers, W. J. Stewart, Johnson, Robertson, Baker, White, Dyson, Leith, Sayre, Hawley, Rishel, Clancy, Casper, and Dr. M. R. Trumbower, State Veterinarian, a visitor. The banquet was very enjoyable, both the service and menu were excellent, and all present enjoyed themselves exceedingly. A toast was given to the Chicago Veterinary Society for its growth and prosperity, in which all heartily concurred.

After the banquet it was moved and seconded to dispense with roll-call and the minutes of the previous meeting, as the hour was late, and that the President call upon Dr. L. A. Merillat for the continuation of his paper on dentistry. This paper was listened to with strict attention. It was instructive as well as interesting, and it met with the hearty applause of all present on its completion.

Dr. Merillat, in this paper, dealt with the diseases of the horse's teeth and the mode of treatment of the affections, describing the entire surgical operations incidental to the treatment and the instruments employed therein, a great deal of which was new to many of the members present. The paper was followed by some discussion and some remarks by prominent veterinary dentists. Those taking active part in the discussion of the paper were Drs. Hughes, Schwarzkopf, E. L. Quitman, P. Quitman, Flowers, Sayre, White, and Casper. On motion, the discussion was closed. A few remarks were made by the President, after which the applications for membership of Dr. Frank Allen, graduate of the American Veterinary College, and Dr. David Kermath, graduate of the Chicago Veterinary College, were offered to the board of censors for their approval. Upon the approval of the board they were both elected to membership by acclamation.

On motion, adjournment.

LAWRENCE CAMPBELL,
Secretary.

---

Dr. Edington, the colonial bacteriologist in South Africa, claims to have discovered the microbe of rinderpest.

